# Selective assembly of Au-Fe_3_O_4_ nanoparticle hetero-dimers

**DOI:** 10.1007/s00604-015-1571-z

**Published:** 2015-07-28

**Authors:** Melissa R. Dewi, Geoffry Laufersky, Thomas Nann

**Affiliations:** ARC Centre of Excellence in Convergent Bio-Nano Science and Technology, Ian Wark Research Institute, University of South Australia, Adelaide, SA 5095 Australia

**Keywords:** Gold nanoparticles, Iron oxide nanoparticles, Dimers, Hetero-dimers, Mono-functionalisation

## Abstract

Hetero-dimeric magnetic nanoparticles of the type Au-Fe_3_O_4_ have been synthesised from separately prepared, differently shaped (spheres and cubes), monodisperse nanoparticles. This synthesis was achieved by the following steps: (a) Mono-functionalising each type of nanoparticles with aldehyde functional groups through a solid support approach, where nanoparticle decorated silica nanoparticles were fabricated as an intermediate step; (b) Derivatising the functional faces with complementary functionalities (e.g. amines and carboxylic acids); (c) Dimerising the two types of particles via amide bond formation. The resulting hetero-dimers were characterised by high-resolution TEM, Fourier transform IR spectroscopy and other appropriate methods.

**Electronic supplementary material:**

The online version of this article (doi:10.1007/s00604-015-1571-z) contains supplementary material, which is available to authorized users.

**Graphical Abstract:**
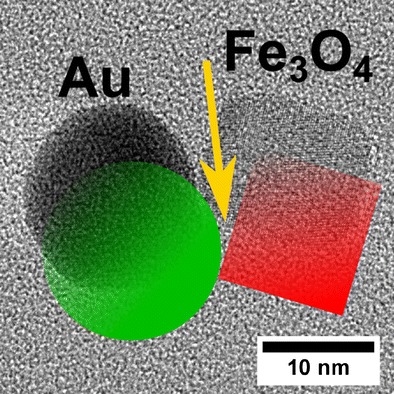
Nano-LEGO: Assembling two types of separately prepared nanoparticles into a hetero-dimer is the first step towards complex nano-architectures. This study shows a solid support approach to combine a gold and a magnetite nanocrystal.

## Introduction

Sketches and simulations of complex nano-machines have been published since the advent of nano-sciences in the early ‘90s. However, the covalent coupling of two different, separately synthesised nanoparticles to form a simple hetero-dimer has turned out to be extremely difficult. In this article, we show a straightforward and generic strategy to accomplish this initial step into nano-architecture design by extending a solid support synthesis method we have developed earlier for the preparation of homo-dimers [1].

The synthesis of high-quality nanocrystals (or nanoparticles) has reached a level where monodisperse and well-defined particles can be made of nearly any material. This includes metallic, semi-conducting and magnetic nanoparticles [2], which have been synthesised and used for numerous (bio-)analytical applications such as, magnetic contrast enhancement and sensing [3–5], bio-imaging [3, 6, 7], fluorescent marking [8, 9], hyperthermia [10, 11], and catalysis [12]. Interestingly, some analytical methods are based on the dimerisation or controlled coagulation of individual nanoparticles [13, 14]. It is known that the properties and performance of nanoparticles are strongly affected by the shape, size, crystalline structure, as well as the monodispersity of the materials [15–17]. Although it is possible to tune the properties of nanoparticles by modifying these attributes, there are limits in engineering chemical and physical properties. To overcome these restrictions, seed-mediated growth methods have been developed for a number of materials, resulting in multi-functional particles [18, 19]. While these techniques are advantageous, they are severely limited in the choice of materials due to the degree of chemical compatibility required for the techniques (e.g. lattice mismatch, redox behaviour, and defect formation). The covalent linkage of two different nanoparticles constitutes the first step in extending the range of engineer-able properties and paves the road for higher complexity nano-systems.

**Scheme 1:**
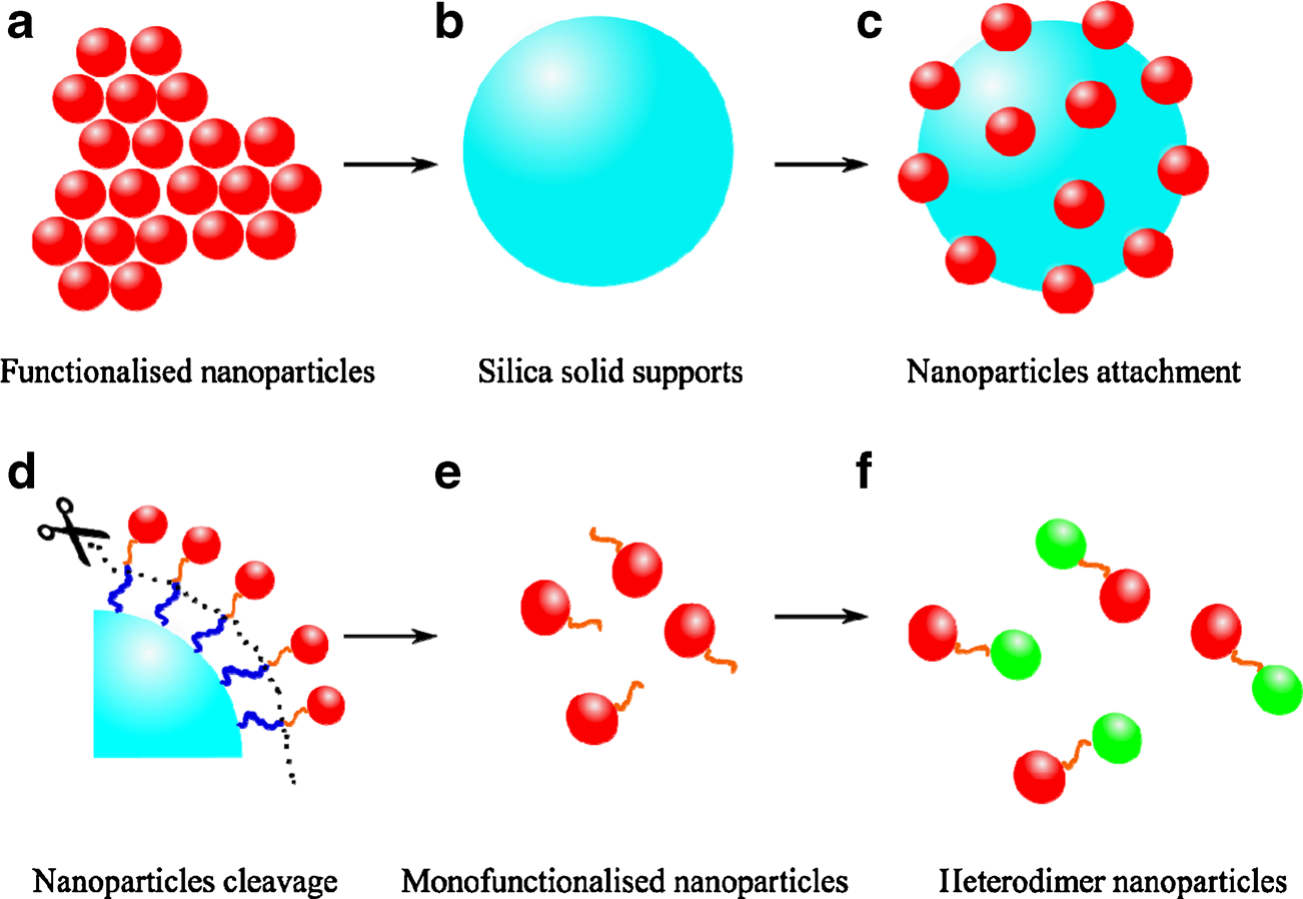
An illustration of nanoparticles monofunctionalisation via the solid support approach

Mono-functionalisation allows nanoparticle manipulation at the molecular level by precisely creating a single functionally active ‘spot’ on the nanoparticle surface consisting of just a few functional groups. Although we demonstrate this strategy only for the example of gold and magnetite nanoparticles (Fe_3_O_4_), we expect it to be applicable generally (Scheme **1**).

Our method is substantially different from the seed-mediated methods mentioned above and biomolecule supported strategies (for example using deoxyribonucleic acid (DNA)) that led to controlled nanoparticle assemblies in the past [20, 21]. The mono-functionalisation strategy used in this work is grounded in modifying nanoparticles selectively at their point of contact to a much larger surface [1, 22, 23]. This strategy follows a similar approach to Merrifield peptide synthesis, thus Merrifield resin [24, 25] and a range of other commercial supports (such as polymer Wang resin [26]) were considered initially. However, none of the commercial supports were suitable for our strategy, as it was found that they tend to wrap the nanoparticles, impeding mono-functionalisation, and making it difficult or impossible to separate from the nanoparticles after the reaction. Silica nanoparticles (SiO_2_ NPs) were selected as a means of providing this rigid, stable, and high-surface area structural support and were successfully used for a different type of mono-functionalisation earlier [1].

## Results and discussion

Silica nanoparticles with an average diameter of about 50 nm were synthesised via the Stöber method [27–30] (a size histogram is available in the supporting information Figure S4). The silica particles were subsequently aminated with 3-aminopropylethoxysilane (APTES) and functionalisatized with a cleavable linker via ethyl (dimethylaminopropyl)carbodiimide / N-hydroxysuccinimide (EDC/NHS) conjugation. Tartaric acid (C_4_H_6_O_6_) was chosen as it can be cleaved by oxidation at its vicinal alcohol groups using sodium periodate (NaIO_4_) (supporting information, Figure S5). The dually carboxylate-terminated molecule also allows for further functionalization via EDC/NHS peptide bond formation.

**Fig. 1:**
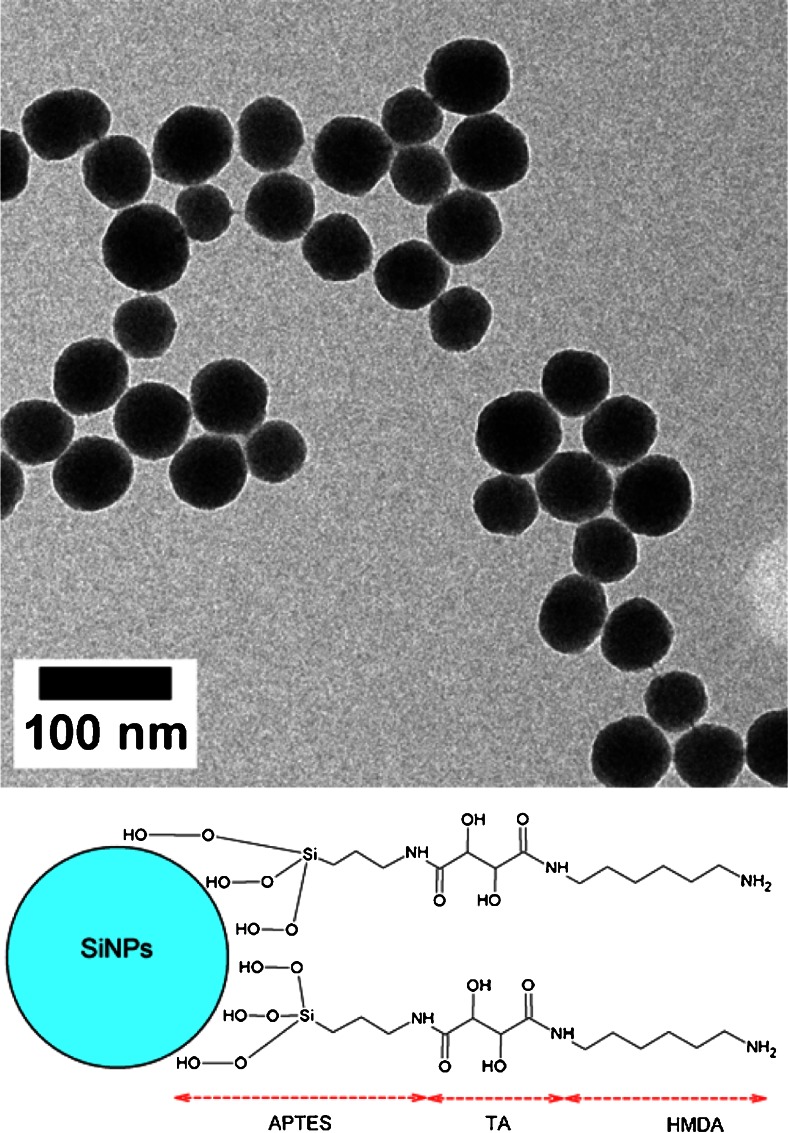
Top: transmission electron micrograph (TEM) of silica nanoparticles. Bottom: schematic representation of the cleavable linker and its components

Finally, hexamethylene diamine (HMDA) was chosen to terminate the surface functionalization to allow subsequent coupling of carboxylated nanoparticles. Figure 1 shows a transmission electron micrograph (TEM) of typical Stöber-silica nanoparticles (top) and the chemical formula of the complete linker (bottom).

The resulting functionalised silica nanoparticles were analysed by means of dynamic light scattering (DLS) and TEM (see Fig. 1, top) to monitor particle integrity. Fourier transform infrared spectroscopy (FTIR) confirmed the final linker had the desired structure as depicted above (details of the synthesis procedures and analytical data can be found in the supporting information).

**Fig. 2:**
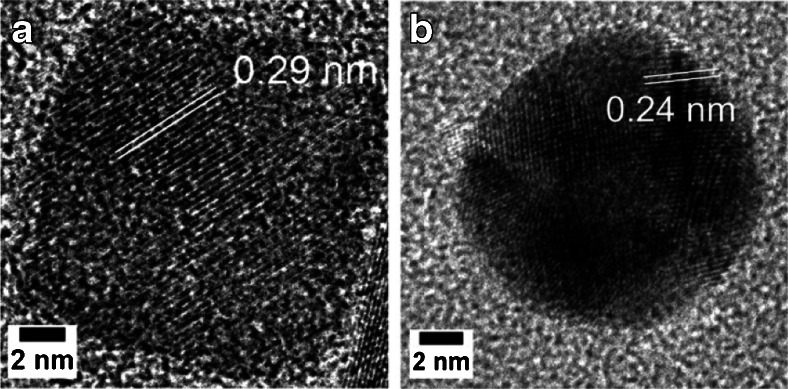
**a** HR-TEM of oxalic acid coated Fe_3_O_4_ nanoparticles; **b** HR-TEM of 3-MPA coated gold nanoparticles

Gold and Fe_3_O_4_ nanoparticles were selected for dimerization, as these nanoparticles have well-established synthetic routes, heavily-studied chemical and physical natures, and very different surface chemistries. Spherical gold nanoparticles with an average diameter of about 11 nm were synthesised by a method previously published by Zheng et al. [31]. The original cetyltrimethylammonium bromide (CTAB) ligands were exchanged against 3-mercaptopropianic acid (3-MPA) using a published method [32]. Cube shaped Fe_3_O_4_ nanoparticles with an edge length of approximately 11 nm were synthesised through thermal decomposition of iron oleate, followed by surface ligand exchange against oxalic acid as reported previously [17]. This resulted in two different types of nanoparticles, which were both carboxylated on their surface (Fig. 2).

**Fig. 3:**
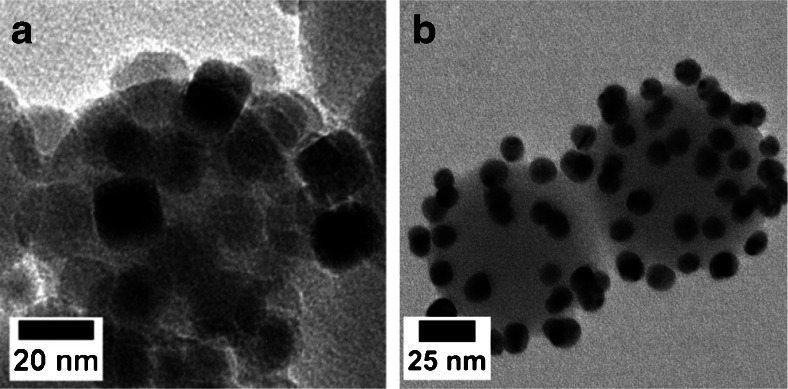
**a** Cube-shaped Fe_3_O_4_ nanoparticles on silica; **b** Spherical gold nanoparticles on silica

The first step in mono-functionalising the nanoparticles included binding the carboxylated gold and Fe_3_O_4_ particles onto the functionalised silica support. The coupling was done by employing the standard coupling agents EDC/NHS (the reaction scheme is outlined in the supporting information, Figure S10). First EDC was reacted with the carboxylic functional groups of both gold and Fe_3_O_4_ nanoparticles to form an active ester (o-acylisourea active intermediate) as the leaving group for the subsequent reaction. However, these leaving groups are subjected to fast hydrolysis in aqueous solution [33]. NHS was simultaneously introduced to stabilise the intermediate and to decelerate the hydrolysis reaction. Then, the activated carboxylated nanoparticles were reacted with the aminated silica support particles and decorated the surface. Figure 3 shows TEM micrographs of cube-shaped Fe_3_O_4_ (Fig. 3a) and spherical gold nanoparticles (Fig. 3b) bound onto functionalised silica particles as described above.

At this stage, the nanoparticles still bore excess activated carboxylic groups on their surface. It is crucial to passivate these groups prior to cleavage in order to obtain mono-functional nanoparticles. We have explored two options at achieve this: first, reaction of the activated carboxylic groups with ethanolamine. Second, swift cleavage and dimerization before hydrolysis of the intermediate occurred to a significant degree (we will discuss this strategy in more detail later). Both options prepared the nanoparticles for the oxidative cleavage.

**Fig. 4:**
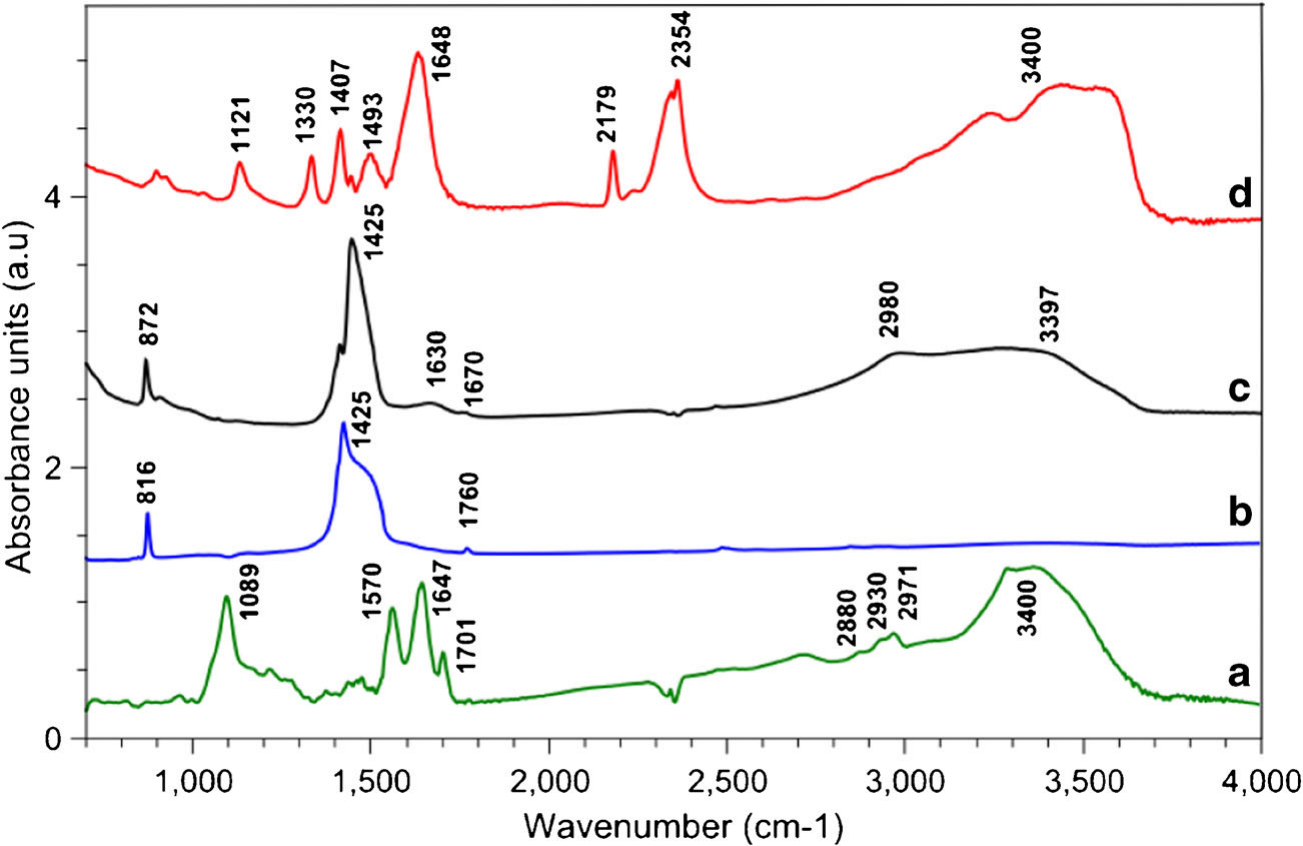
FTIR spectra of Fe_3_O_4_ nanoparticles at various stages of mono-functionalisation: **a** nanoparticles coupled to the solid support; **b** nanoparticles after dissolution of silica particles; **c** mono-functional nanoparticles after oxidative cleavage; **d** mono-functional nanoparticles after reaction with glycine. FTIR spectra of gold nanoparticles is provided in supporting information

After cleavage it was found that it was very difficult to separate the silica nanoparticles from the mono-functionalised gold and Fe_3_O_4_ nanoparticles in dispersion. Therefore, silica nanoparticles were dissolved by addition of 1 M NaOH followed by sonication, which resulted in the fast digestion of the silica particles without disruption of the mono-functionalised nanoparticles. Figure 4 shows the Fourier-Transform Infra-Red (FTIR) spectra of the Fe_3_O_4_ nanoparticles at the different stages of the mono-functionalisation (equivalent spectra for gold particles can be found in the supporting information). Figure 4a depicts the spectrum of the particles bound to the silica solid support. The peaks at 1570, 1647 and 1701 cm^−1^ are indicative for the amide bond that has formed on coupling the nanoparticles to the solid support. After dissolution of the silica, the spectrum (Fig. 4b) is clearly dominated by the peak at 1425 cm^−1^, which can be assigned to the C-H bending vibration of alkanes. It is anticipated that the increased flexibility of the linker by removal of the silica causes this enhancement in signal. Figure 4c shows the FTIR spectrum after oxidative cleavage, where the peak at 1630 cm^−1^ can be assigned to the newly formed aldehyde.

After cleavage, silica dissolution, and purification, both types of nanoparticles bore aldehyde groups clustered in the monofunctionalization ‘spot’ (confirmed by FTIR spectroscopy). In order to form hetero-dimers, the two types of nanoparticles have to be functionalised with different reactive groups. In principle, it would be sufficient to convert the functional spot of one type of nanoparticles to amines and then dimerise two different types by reductive amination (this would also make the passivation step discussed above obsolete). However, for the sake of generality, we also functionalised one type of nanoparticle with carboxylic and the other with primary amine groups for subsequent formation of an amide bond. Mono-functionalised Fe_3_O_4_ nanoparticles were reacted with glycine by reductive amination; the primary amine of glycine reacts with the aldehyde groups of the nanoparticles and forms a Schiff base. Schiff bases are unstable and readily reversed by hydrolysis in aqueous solutions, therefore, they were reduced to secondary amines by addition of sodium cyanoborohydride (NaCNBH_3_) [33]. The spectrum in Fig. 4d confirms the formation of the secondary amine, mainly by the N-H bending vibration at 1493 cm^−1^. Gold nanoparticles were reacted with an excess of ethylenediamine (EDA) in the same manner, which resulted in amine mono-functionalised particles.

Au-Fe_3_O_4_ nanoparticle hetero-dimers were synthesised by dispersing both particles types in the same solution and coupling with the EDC/NHS conjugation method discussed above. Through the conjugation, carboxylate groups of Fe_3_O_4_ nanoparticles were reacted with amine groups of gold nanoparticles and formed amide bonds.

**Fig. 5:**
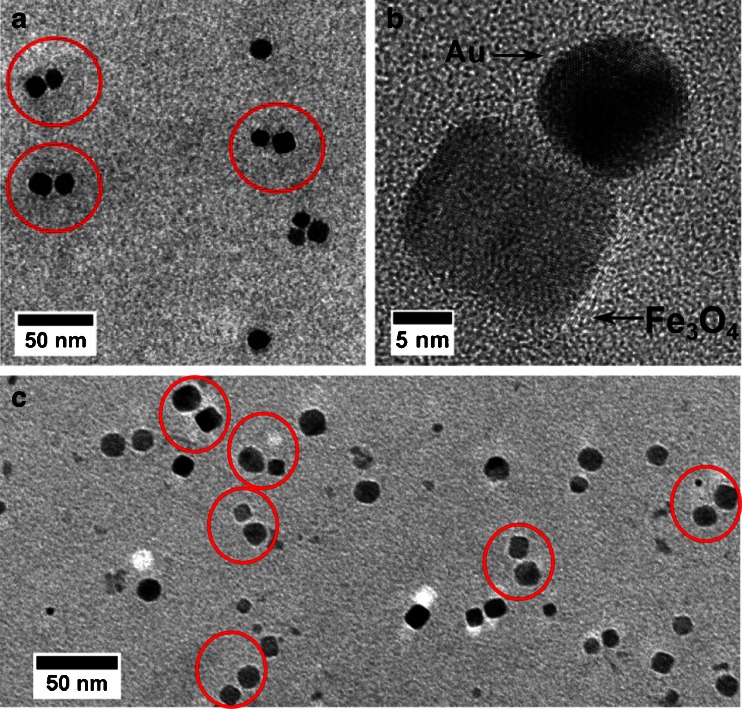
TEM micrographs of **a** overview of Au-Fe_3_O_4_ hetero-dimer nanoparticles, **b** HR-TEM of one Au-Fe_3_O_4_ hetero-dimer and **c** another overview of Au-Fe_3_O_4_ hetero-dimer nanoparticles

**Fig. 6:**
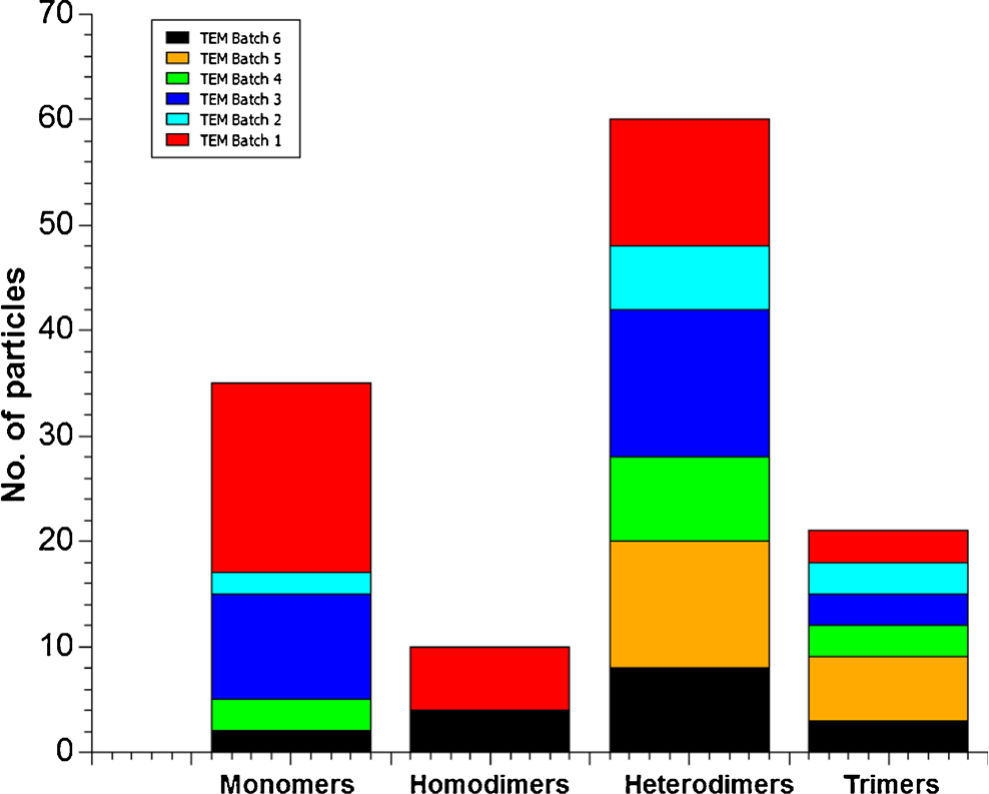
Statistics for heterodimer formation of spherical gold and cube shaped Fe_3_O_4_ nanoparticles

Figure 5 shows an overview of typical TEM images of the Au-Fe_3_O_4_ nanoparticle hetero-dimers for cube shaped Fe_3_O_4_ and spherical gold nanoparticles (Figure S13 in the supporting information shows larger overview images). It was observed that hetero-dimers were formed selectively even though a few monomers, oligomers and homo-dimers are still present. A statistical analysis of several overview images resulted in an approximate yield for dimerisation of about 40 % with more than 90 % of the dimers were hetero-dimers. Some of the undesired structures may have been formed by aggregation rather than controlled coupling (Fig. 6).

**Fig. 7:**
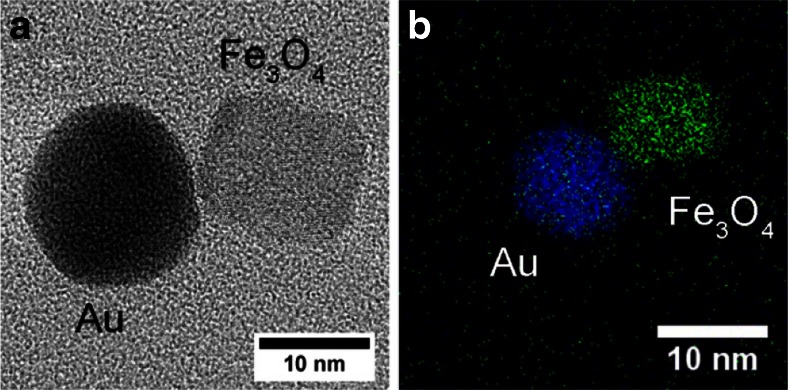
(*Left*) HR-TEM of Au-Fe_3_O_4_ hetero-dimers and (*Right*) Overlay elemental mapping of Au-Fe_3_O_4_ hetero-dimer nanoparticles from STEM

Detailed information on the structure and composition of hetero-dimer nanoparticles has been obtained from high-resolution transmission electron microscopy (HRTEM) images, where one example is shown in Fig. 5b. The HRTEM micrograph clearly shows the lattice fringes of spherical gold and cube-shaped Fe_3_O_4_ nanoparticles. Figure **7** shows another example of an individual hetero-dimer on the left and the elemental mapping of a hetero-dimer using scanning transmission electron microscopy (STEM) on the right.

XRD, UV–vis and further FTIR analysis were carried out to confirm the formation of heterodimer of Au-Fe_3_O_4_ nanoparticles (data are shown in the supporting information).

## Conclusions

In this work, we have synthesised hetero-dimer nanoparticles using a solid support approach by first creating a mono-functional surface for both designated nanoparticles (Fe_3_O_4_ and gold Nanoparticles). We have shown that the attachment of the nanoparticles onto the solid support and passivation steps were crucial as this will dictate whether or not a point of contact (mono-functional group) has been created. We have established a generic way to couple two dissimilar (in regards to their chemical, physical properties and core materials) and separately prepared nanoparticles covalently. The creation of hetero-dimer nanoparticles from simple nanoparticle building blocks represents the first step towards controlled assembly of complex nano-architectures. Furthermore, nanoparticle hetero-dimers have many applications in their own right, for example as multimodal contrast agents for bioimaging.

## Electronic supplementary material

ESM 1 (DOC 4.71 MB)
