# Supplementary material for: Selective assembly of Au-Fe3O4 nanoparticle hetero-dimers
Source: Mikrochim Acta. 2015 Jul 28;182(13-14):2293–8. doi: 10.1007/s00604-015-1571-z (PMC4569660; doi:10.1007/s00604-015-1571-z)
Supplement: Supplementary file 1 — (DOC 4.71 MB) [file 604_2015_1571_MOESM1_ESM.doc]

**Electronic Supplementary Material**

**Selective assembly of Au-Fe_3_O_4_ nanoparticle hetero-dimers**

Melissa R. Dewi,^a^ Geoffry Laufersky^a^ and Thomas Nann^*a^

*^a^ ARC Centre of Excellence in Convergent Bio-Nano Science and Technology. Ian Wark Research Institute, University of South Australia, Adelaide, SA 5095, Australia*

*Corresponding author, E-mail: [thomas.nann@unisa.edu.au](mailto:thomas.nann@unisa.edu.au); Phone: +61 8 8302 5369

1. **Experimental section**

All chemicals have been purchased from Sigma-Aldrich and used without further purification (www.sigma-aldrich.com).

- 1. **Synthesis of silica nanoparticle (SiNPs) solid supports**

Functionalised silica nanoparticles (SiNPs) were synthesized based on the method we used from our previous work^1^ with an additional modification as described below:

**Preparation of HMDA-functionalised silica nanoparticles**. HMDA-functonalised silica nanoparticles were prepared using the same standard conjugation method utilising EDC and NHS as above. Taking into consideration that each -COOH molecule from the carboxylated SiNPs reacts with one amine from HMDA in order to form a peptide bond, a 1:1 molar ratio was applied. In conjunction with this step, a 10-fold excess of EDC and NHS were quickly added into the reaction mixture. The mixture was vigorously stirred at RT for 3 hours. HMDA functionalised SiNPs were then purified by centrifugation to remove excess conjugation agents, redispersed in water and this process was repeated three times at 19,000 rcf for 15 minutes each. Finally, the nanoparticles were homogenously dispersed in 10 mL water for storage.

- 1. **Synthesis of Fe_3_O_4_ and Au nanoparticles**

To synthesize spherical Au and cubic Fe_3_O_4_ nanoparticles nanoparticles, we used a synthesis method that we have published before.^2^

- 1. **Conjugation of Au and Fe_3_O_4_ nanoparticles onto SiO_2_ solid supports**

NPs (Fe_3_O_4_ and Au) were surface functionalised with oxalic acid (C_2_H_2_O_4_) and 3-mercaptopropionic acid/ 3-MPA (HSCH_2_CH_2_CO_2_H) respectively to feature carboxylate functional groups (-COOH) whilst SiNPs solid support templates feature hexamethylene diamine/HMDA (C_6_H_16_N_2_) to expose primary amine groups.

To attach the functionalised nanoparticles onto the SiNPs, 100 µL of HMDA functionalised SiNPs were mixed with 100 µL (50mg mL^-1^) of Au-MPA NPs and the mixture was diluted by adding 4.8 mL water. Into the system, 0.3 mmol of NHS and EDC were quickly added. The mixture was then reacted at RT for 3 hr. After reaction, the resulting mixture was centrifuged at 19,000 rcf for 15 minutes and washed twice with water. An identical procedure was also employed to Fe_3_O_4_ NPs. After purification, both SiNPs-Au nanostructures and SiNPs- Fe_3_O_4_ nanostructures were redispersed in 1mL water.

|  |
| --- |
|  |
| **Figure S1:** The preparation of the “Strawberry” nanostructures via SiNPs solid support attachment to the functionalised NPs (Au and Fe_3_O_4_ NPs). This process is represented in Schematic 5.1 A-D. |

- 1. **Nanoparticle monofunctionalisation**

As depicted in Scheme 1, the obtained SiNPs-Au and SiNPs- Fe_3_O_4_ nanostructures were subjected to be monofunctionalised. The monofunctionalisation procedure consists of two main stages:

1. **Oxidative cleavage of diols by Sodium Periodate (NaIO_4_)**

To segregate the passivated Au and Fe_3_O_4_ NPs from the SiNPs solid support templates, 2 mL NaOH (1M) was added into 1 mL SiNPs-Au nanostructures solution. The resulting mixture was then sonicated for 30 minutes to dissolve the SiNPs. After sonication, the NP mixture was centrifuged at 19,000 rcf for 10 minutes and the supernatant was discarded. The resulting precipitate was re-dispersed in 1 mL water. Furthermore, 1 mL mixture containing Au NPs after separation from solid support was then mixed with a solution of 326 µL of NaIO_4_ (10mg mL^-1^) and reacted for 30 minutes at RT without the presence of light (covering the vial with aluminium foil to avoid light penetration to the system). After reaction, the resulting mixture was then centrifuged at 19,000 rcf for 10 minutes and the precipitation was re-dispersed in 1 mL water. The same procedure was used to cleave the passivated Fe_3_O_4_ NPs from the solid supports.

|  |
| --- |
|  |
| **Figure S2:** The separation of SiNPs from the respective NPs via oxidative cleavage of diols (L-tartaric acid) by NaIO_4_. Solid supports were removed from the system by addition of 1M NaOH. |

1. **Reductive amination**

Both monofunctionalised Au and Fe_3_O_4_ NPs feature aldehyde functional groups on their surfaces as a result of the oxidative cleavage by NaIO_4_. In order to from heterodimers, the two types of nanoparticles have to be further functionalised with different surface functional groups via reductive amination.

For the Au NPs, into the 1mL monofunctionalised AuNPs, 1mL NaCl (0.15 M) and 1 mL HMDA (10 mg mL^-1^) and 30 µL of NaCNBH_3_ were added and the mixture was reacted for 2 hours to achieve the complete reductive amination. After reaction, the mixture was then centrifuged for 15 minutes at 19,000 rcf and the resulting precipitate was re-dispersed in 1mL water. For Fe_3_O_4_ NPs, into the 1 mL of cleaved Fe_3_O_4_ NPs, 1 mL NaCl (0.15 M) and 1 mL glycine (10 mg mL^-1^) and 30 µL of NaCNBH_3_ were added and reacted for 2 hours to achieve complete reductive amination. After reaction, the mixture was then centrifuged for 15 minutes at 19,000 rcf and the resulting precipitate was re-dispersed in 1 mL water.

|  |
| --- |
|  |
| **Figure S3:** The reductive amination of monofunctionalised Fe_3_O_4_ NPs and Au NPs with glysine and ethyleneamine respectively to form different functional groups on both NPs to allow heterodimerisation. |

- 1. **Nanoparticle heterodimerisation**

The resulting mixture of 1 mL Au NPs from the reductive amination stage was mixed with 1 mL of Fe_3_O_4_ NPs from reductive amination. This mixed solution was diluted to 4 mL volume with water. Into the system 0.1 mmol of EDC and NHS were quickly added and let the mixture stir for 3 hours at RT. Next, the resulting mixture was then centrifuged at 19,000 rcf for 15 minutes and the resulting precipitate was washed with water for twice and finally redispersed in 2 mL water for storage.

**Transmission Electron Microscope (TEM).** TEM imaging was carried out by using a FEI Titan 80-300 (abberation corrected TEM) operated at 300kV and JEOL 2100F operated at 200 kV. TEM samples were prepared by placing a small drop of solutions on a typical 3 mm TEM carbon holey film on a copper grid supplied by Agar Scientific.

**Dynamic Light Scattering (DLS).** The particles size determination was carried out at 25^o^C by using a Zetasizer-Nano instrument from Malvern, UK.

**X-Ray Diffraction (XRD).** X-ray diffraction has been used to determine the crystalline properties of the nanocubes. The samples were analysed using Scintag ARL X'tra diffractometer and CuKα radiation. XRD traces were collected between 2^o^ and 90^o^ 2θ at 0.02^o^ intervals at rate 0.08^o^ per minute.

**Fourier Transform Infrared (FTIR).** Infrared spectra analysis of both pre and post ligand exchange of the nanocubes were carried out by using the transmission mode of VERTEX 70 FT-IR Spectrometer with HYPERION 3000 FT-IR Microscope from Bruker Optics. FTIR samples were prepared by placing a small drop of nanoparticles solution on a 2 cm x 2 cm of reflective substrate (cut silicon wafer). The sample then dried in a vacuum desiccator to create a very thin layer on the silicon wafer prior to the measurement.

**Ultraviolet-visible spectroscopy (UV-VIS).** UV-Vis spectra were recorded with a Cary 300 UV-Vis spectrometer with UV-Vis quartz cuvette.

1. **Silica solid support characterisation**

**
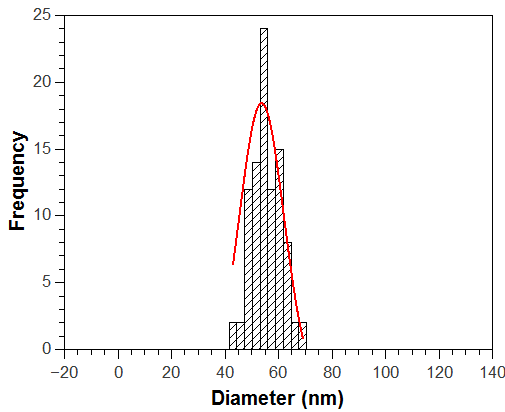
**

| 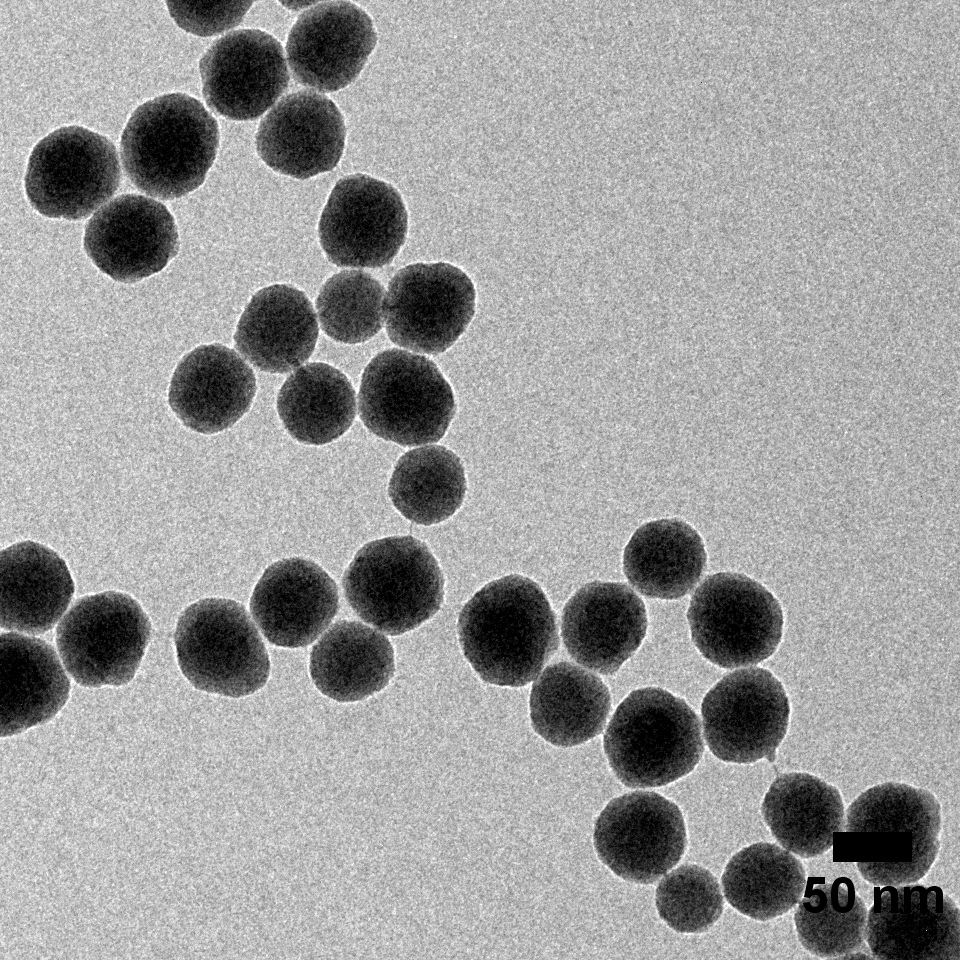 |
| --- |
| Figure S4: (A) TEM image of HMDA-TA-APTES-SiNPs, (B) The particle histogram of the HMDA-TA-APTES SiNPs from TEM micrographs measured and analysed by ImageJ. |

| 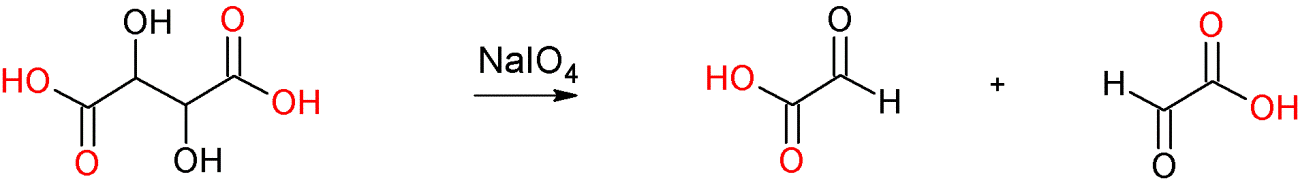 |
| --- |
| Figure S5: Tartaric acid molecules contain hydroxyl functional groups attached to the adjacent carbon atoms (left reactant). The oxidation mechanism by sodium periodate (NaIO_4_) to cleave the bond in between the hydroxyl groups to produce aldehydes (bottom), which aids the monofunctionalisation step. |


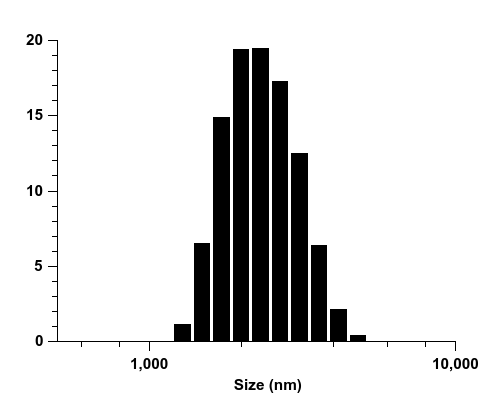


Figure S6: Size distribution measured by number of the DLS for HMDA-TA-APTES SiNPs.

**C**


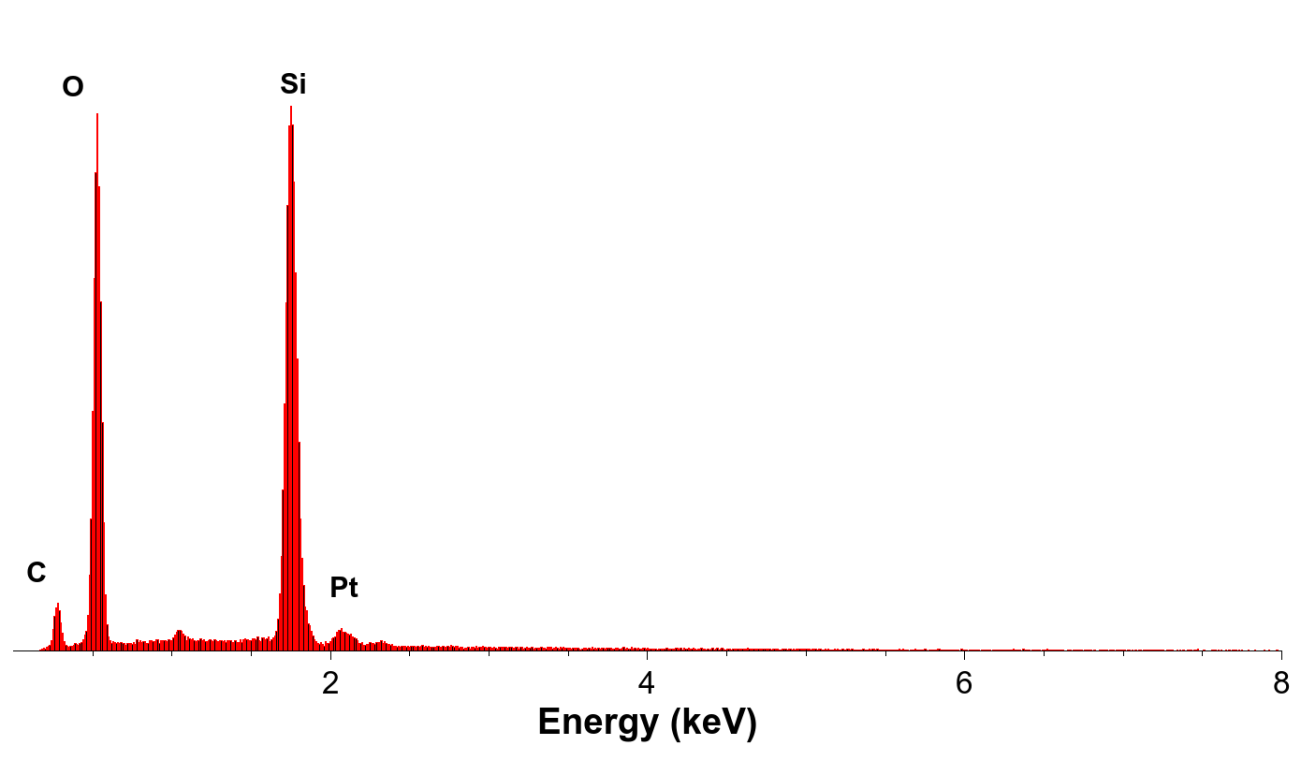

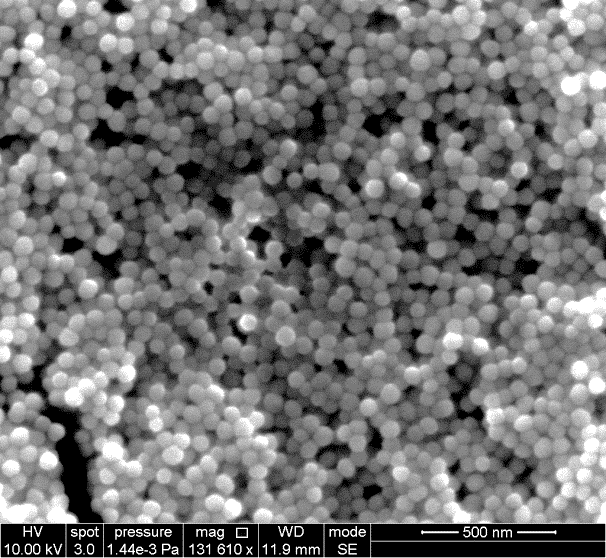


**C**


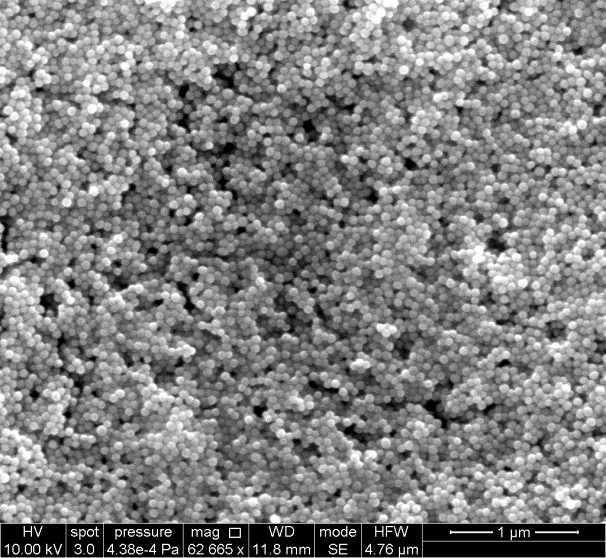


**A**


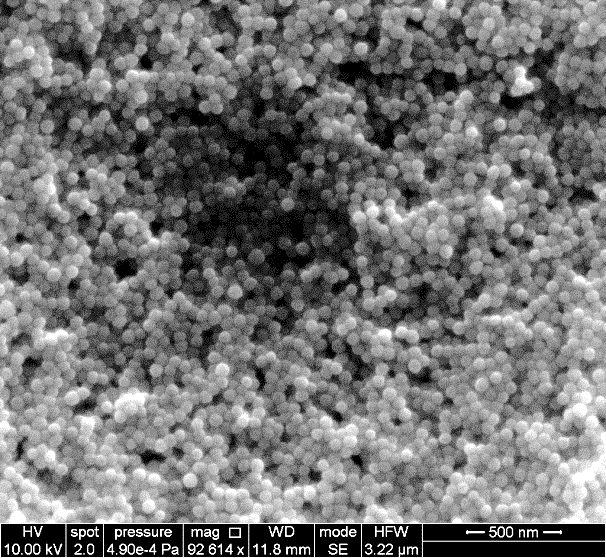


**B**

**A**

**B**

**C**

Figure S7: The SEM images of monodisperse spherical SiNPs (A) 62k magnification, (B) 90k magnification and (C) 130k magnification. The confirmation of Si elemental composition has been confirmed with EDX as depicted in (C).

To confirm the successful surface functionalisation of SiNPs, FTIR analysis has been carried out to detect the functional groups attached to the SiNP surfaces.

| 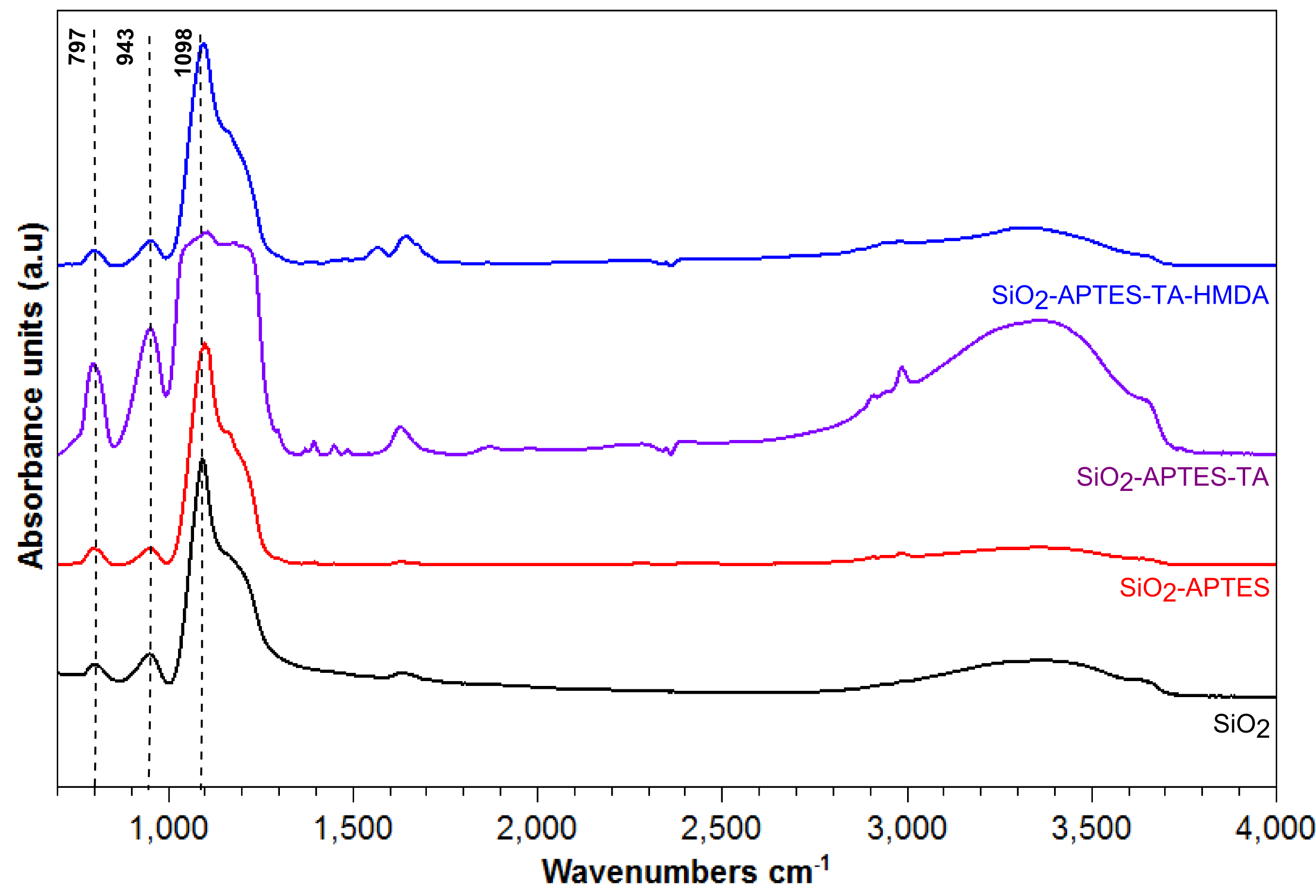 |
| --- |
| Figure S8: The full FTIR spectra for the un-modified and modified silica nanoparticles. Changes of the peaks show the successful grafting of the functional groups onto the silica nanoparticles surface. |

The functional groups of the SiNPs were identified by comparing the FTIR spectra of each modification step of the SiNPs. The black line from Figure 4.13 of the FTIR spectra represents the un-modified colloidal SiNP spectra. The three typical bands at 797 cm^-1^, 943 cm^-1^, and 1094 cm^-1^ are found in each spectrum and were assigned to the symmetric vibration of Si-O, asymmetric vibration of Si-OH and asymmetric vibration of Si-O respectively. For the colloidal, un-modified SiNPs the band at 1632 cm^-1^ arises due to scissor bending vibration of the molecular water absorbed on the surface of the SiNPs (Figure 13). In conjunction to this band, molecular water also shows a quite prominent peak at 3400 cm^-1^ due to the O-H stretch in H-bound water.

**A**

**B**


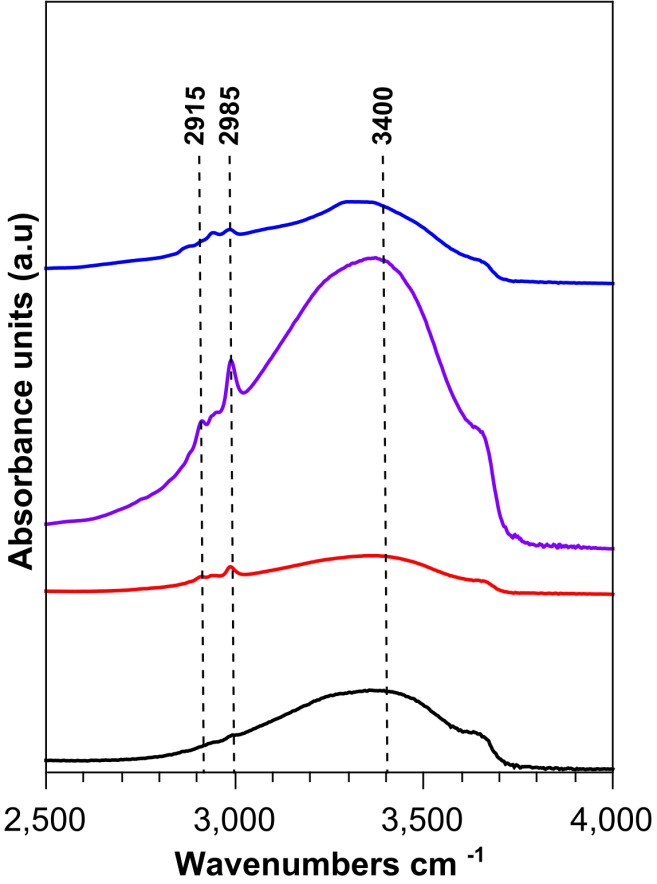

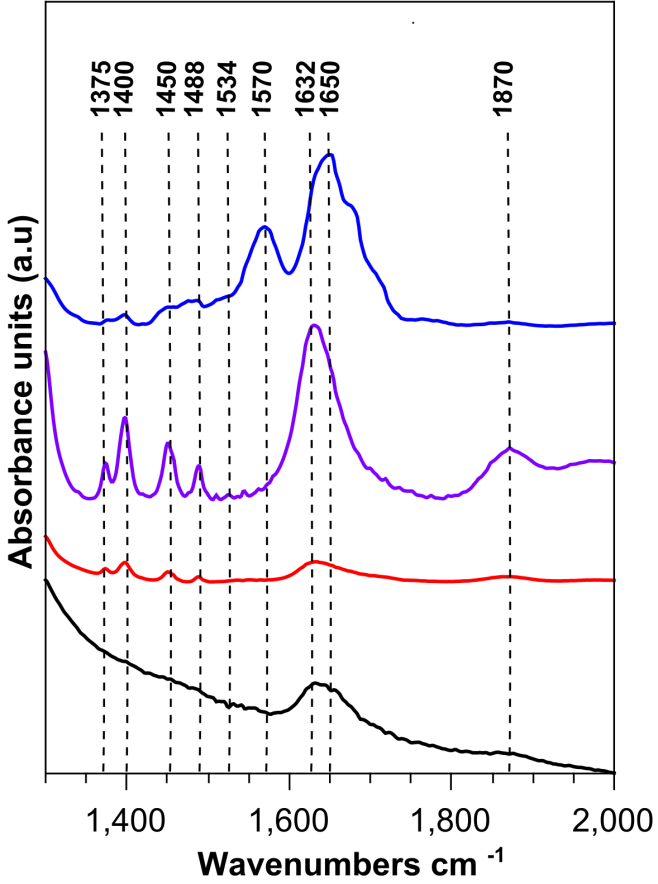


|  |  |
| --- | --- |
| Figure S9: (A) FTIR spectra in the range of 1200 cm^-1^ – 2000 cm^-1^ ; (B) FTIR spectra in the range of 2500 cm^-1^ – 4000 cm^-1^ (please refer to Figure 4.14 for the spectra color coding). | |

The reaction of the SiNPs with APTES resulted in spectral changes by the appearance of several low intensity peaks. New peaks at the ranges of ~2880-2973 cm^-1^ and ~1375-1488 cm^-1^ are due to the Si-CH_2_ stretching and bending vibration respectively. A new peak also appears at ~1534 cm^-1^ due to NH_2_ bending which indicates the successful conjugation of APTES onto SiNPs. In addition, the conjugation of APTES onto the SiNPs surface also resulted in a significant reduction of the intensity of the O-H streching and the scissor bending vibration of the molecular water at 3400 cm^-1^ and 1632 cm^-1^ respectively. Furthermore, a wider band at 3400 cm^-1^ due to the overlapping of asymmetric stretching vibrations of N-H bonds and C-H vibration with siloxane and silanol groups has also been observed as the consequence of having APTES conjugated to the SiNPs surface.

Upon functionalisation with tartaric acid, the amide bond formation is indicated by intensity changes on several peaks. A significant increase at 1633 cm^-1^ shows the C=O stretching vibration of the amide bond formation as the result of SiO_2_-APTES conjugation with tartaric acid. Furthermore, peaks in the range of ~2915 – 2985 cm^-1^ and 3400 cm^-1^ have significantly increased and can be attributed as the existence of C-H stretching vibration and the overlapping vibrations of N-H bending from the amide bonds and O-H stretching vibration from the COOH functional groups respectively. The conjugation of tartaric acid to the SiO_2_-APTES can also be indicated from the increased intensity of the peaks in the range of 1400 – 1440 cm^-1^, which attributes to the O-H bending vibrations.

The conjugation of HMDA onto SiO_2_-APTES-TA was confirmed by the appearance of two prominent peaks at 1570 cm^-1^ and 1650 cm^-1^ which represents the N-H bending vibrations and C=O stretching vibrations of the amide bonds respectively. A less intense peak at 3400 cm^-1^ was also observed as the consequence of having no COOH groups and this peak shows N-H stretching vibrations of the conjugated HMDA to the tartaric acid functionalised SiNPs.

1. **Mechanism of EDC/NHS conjugation**

| **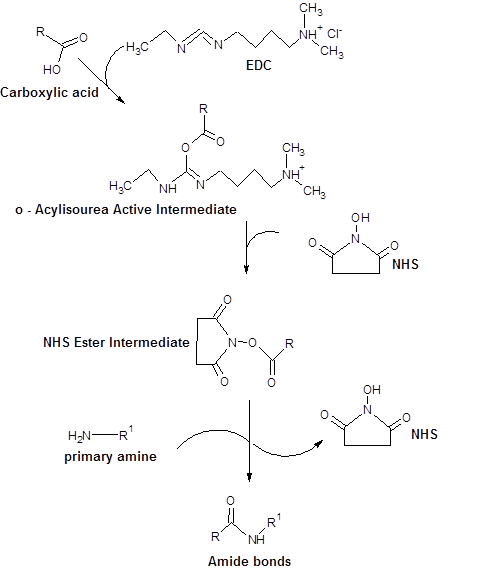** |
| --- |
| Figure S10: The mechanism of the EDC/NHS conjugation. By introducing the NHS into the system, the yield of the amide bond formation increases due to the NHS intermediate having slower hydrolysis rate than the active intermediate from EDC by itself. |

1. **FTIR spectra of heterodimer nanoparticles**

To confirm the bonding within the coupled Au-Fe_3_O_4_ NPs (covalent linkage), FTIR analysis was carried out and it is shown in Figure S12. This spectrum shows the formation of the peptide (amide) bonds after the Au and Fe_3_O_4_ conjugation. The attachment of the nanoparticles onto the solid support was successfully carried out via the peptide bonds as the result of the EDC/NHS conjugation between the functionalised Au and Fe_3_O_4_ NPs. This was confirmed by the appearance of the characteristic bands at 1570 cm^-1^, 1647 cm^-1^,1701 cm^-1^, which were assigned to the N-H bend, C=O stretch, and C=O stretch respectively of the amide bond formation. The prominent peak at 1098 cm^-1^ indicates the existence of Si-O-Si from the SiNP solid supports. The presence of a peak at 3400 cm^-1^ is due to the O-H stretch in H-bound water (Table S1). The SiNP solid supports separation resulted in spectral changes by the absence of Si-O-Si peak at 1098 cm ^-1^, this is obvious that the SiNPs have left the system. In addition, several peaks at 816 cm ^-1^, 1425 cm ^-1^, 1745 cm ^-1^ and 2800-2950 cm ^-1^ can be assigned as N-H wag of the secondary amines from the peptide bonds, C-H bend, C=O stretch and C-H stretch respectively. It is crucial to ensure the leaving of SiNPs solid supports from the system and this has been proved by the disappearance of Si-O-Si peak. Upon SiNPs dissolution, the swift cleaving via periodate oxidation was carried out to cleave the carbon-carbon of the adjacent diols of the tartaric acid to form aldehyde groups. The two prominent peaks to indicate the presence of aldehydes are formed which are at ~1670 cm ^-1^ and ~2980 cm ^-1^ which can be assigned as the C=O stretch and C-H aldehyde stretch respectively. Upon cleaving, the Au and Fe_3_O_4_ NPs were further functionalised via reductive amination to create amine and carboxylate functional groups on the surface of Au NPs and Fe_3_O_4_ NPs respectively. From the reductive amination reaction, the appearance of several peaks for amine monofunctionalised AuNPs are presence at 1566 cm^-1^ and 1625 cm ^-1^, which can be assigned as the N-H bends of the primary amines. Two peaks are presence at 3210-3380 cm ^-1^ represents the N-H peaks. In addition, the Fe_3_O_4_ NPs reductive amination reaction show several peaks at 1330 cm ^-1^,1407 cm ^-1^ ,1493 cm ^-1^ and 1690 cm ^-1^ which can be assigned as C-O stretch, O-H bend, N-H bend (secondary amine) and C=O stretch respectively.

An equivalent spectrum for the Au nanoparticles at the different stages of the functionalisation is shown in Figure S12 E-H. Figure S12 depicts the spectrum of the particles bound to the silica solid support. The peaks at 1571 cm^-1^, 1651 cm^-1^ and 1709 cm^-1^ are indicative for the amide bond that has formed on coupling of the nanoparticles to the solid support. After dissolution of the silica, spectrum S11.F was obtained.

| 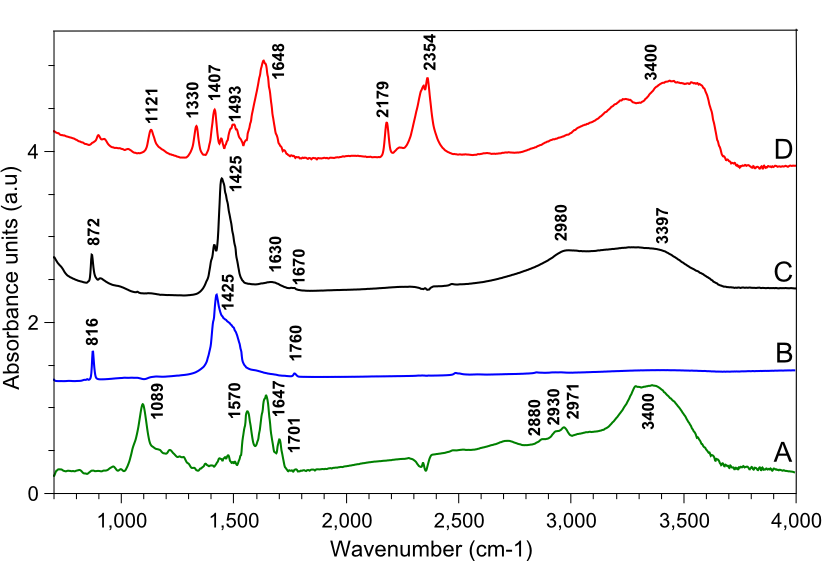 |
| --- |
| 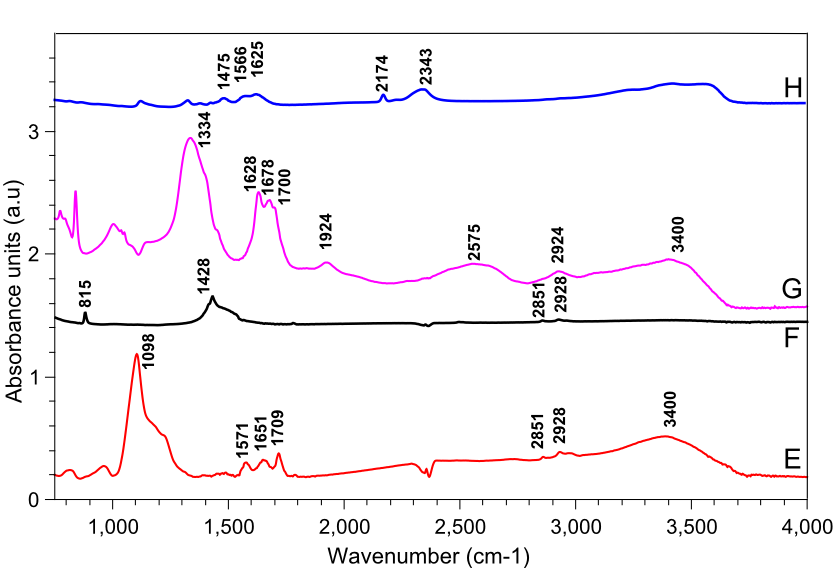 |
| **Figure S11:** FTIR spectra of treated cubic Fe_3_O_4_ (A-D) and spherical Au (E-H). |

**Table S1:** Table of IR absorptions for representative functional groups.

| Iron Oxide (Fe_3_O_4_) NPs | **Label** | **Treatment** | **Wavenumber (cm^-1^)** | **Assignment** |
| --- | --- | --- | --- | --- |
|  | **A (green)** | Fe_3_O_4_ functionalised NPs attachment to the SiNPs solid supports (Strawberry NPs). | 1098 | Si-O-Si |
|  |  |  | 1570 | N-H bend (amides) |
|  |  |  | 1647 | C=O stretch (amides) |
|  |  |  | 1701 | C=O stretch (amides) |
|  |  |  | 2800-2950 | C-H stretch |
|  |  |  | 3400 | O-H stretch |
|  | **Ba(blue)** | SiNPs solid support separation treatment by addition of 1M of NaOH. | 816 | N-H wag (secondary amines) |
|  |  |  | 1425 | C-H bend (alkanes) |
|  |  |  | 1745 | C=O (amides) |
|  |  |  | 2800-2950 | C-H stretch |
|  | **C (black)** | The cleaving of tartaric acid. | 872 | N-H wag (secondary amines) |
|  |  |  | 1425 | C-H bend (alkanes) |
|  |  |  | 1630 | C=O stretch (aldehyde) |
|  |  |  | 1670 | C-O stretch (amide) |
|  |  |  | 2980 | C-H stretch |
|  |  |  | 3397 | O-H stretch |
|  | **D (red)** | Reductive amination of the cleaved Fe_3_O_4_ NPs with glycine. | 1121 | C-O stretch (COOH) |
|  |  |  | 1330 | CH_2_ deformation |
|  |  |  | 1407 | O-H bend (COOH) |
|  |  |  | 1493 | N-H bend (secondary amine) |
|  |  |  | 1690 | C=O stretch (COOH) |
|  |  |  | 2179 | B-H stretch (NaBH_3_CN) |
|  |  |  | 2354 | B-H stretch (NaBH_3_CN) |
|  |  |  | 3400 | O-H stretch (COOH) |
| Gold (Au) NPs | **E**  **(red)** | Au functionalised NPs attachment on to the SiNPs solid supports (Strawberry NPs) | 1098 | Si-O-Si |
|  |  |  | 1571 | N-H bend (amides) |
|  |  |  | 1651 | C=O stretch (amides) |
|  |  |  | 1709 | C=O stretch (amides) |
|  |  |  | 2851 | C-H stretch |
|  |  |  | 2928 | C-H stretch |
|  |  |  | 3400 | O-H stretch |
|  | **F**  **(black)** | SiNPs solid support separation treatment by addition of 1M of NaOH. | 815 | N-H wag (secondary amines) |
|  |  |  | 1428 | C-H bend (alkanes) |
|  |  |  | 2851 | C-H stretch |
|  |  |  | 2928 | C-H stretch |
|  | **G**  **(pink)** | The cleaving of tartaric acid. | 1334 | C-O stretch |
|  |  |  | 1628 | C=O stretch (aldehyde) |
|  |  |  | 1678 | C-O stretch (amide) |
|  |  |  | 1700 | C=O stretch (COOH) |
|  |  |  | 2575 | C-H stretch |
|  |  |  | 2924 | C-H stretch |
|  |  |  | 3400 | O-H stretch |
|  | **H**  **(blue)** | Reductive amination of the cleaved Au NPs with Ethylenediamine. | 1475 | CH2 bend (alkanes) |
|  |  |  | 1566 | N-H bend (primary amines) |
|  |  |  | 1625 | N-H bend (primary amines) |
|  |  |  | 2174 | B-H stretch (NaBH_3_CN) |
|  |  |  | 2343 | B-H stretch (NaBH_3_CN) |
|  |  |  | 3210-3380 | N-H stretch (2 peaks) |

This spectrum is clearly dominated by the peak at 1428 cm^-1^, which can be assigned to the C-H bending vibration of alkanes. It is anticipated that the increased flexibility of the linker by removal of the silica causes this enhancement in signal. Figure S12G shows the FTIR spectrum after oxidative cleavage. The most important difference to spectrum B is the peak at 1628 cm^-1^, which can be assigned to the newly formed aldehyde. Figure S12H shows the FTIR spectrum after reductive amination. Several new peaks at 1566 cm^-1^ ,1625 cm^-1^, 3210-3380 cm^-1^ (two peaks) can be assigned as N-H bend (primary amines), N-H bend (primary amines),and N-H stretch (two peaks) which are indicative for the primary amine bond that has formed upon the reductive amination with ethylenediamine. Further FTIR analysis was carried out to confirm the covalent bond within the Au-Fe_3_O_4_ hetero-dimers. Several peaks for peptide/amide bonds are present at 1517 cm^-1^, 1573 cm^-1^, 1653 cm^-1^ and 3180 cm^-1^, which can be assigned to N-H bend (1^o^), N-H bend, C=O stretch and N-H stretch vibrations of the amide bonds respectively.

1. **Scanning – Transmission Electron Microscope (S-TEM)**

To confirm that the dimerised nanoparticles were constructed from Fe_3_O_4_ NPs and Au NPs, scanning transmission electron microscopy (STEM) based on energy dispersive X-ray spectroscopy (EDS) was used. Figure S13A shows the bright field STEM image which represents the Au-Fe_3_O_4_ heterodimer nanoparticles. The elemental mapping of the blue dots corresponds to the existence of gold element whereas green colour corresponds to the existence of iron element.

| 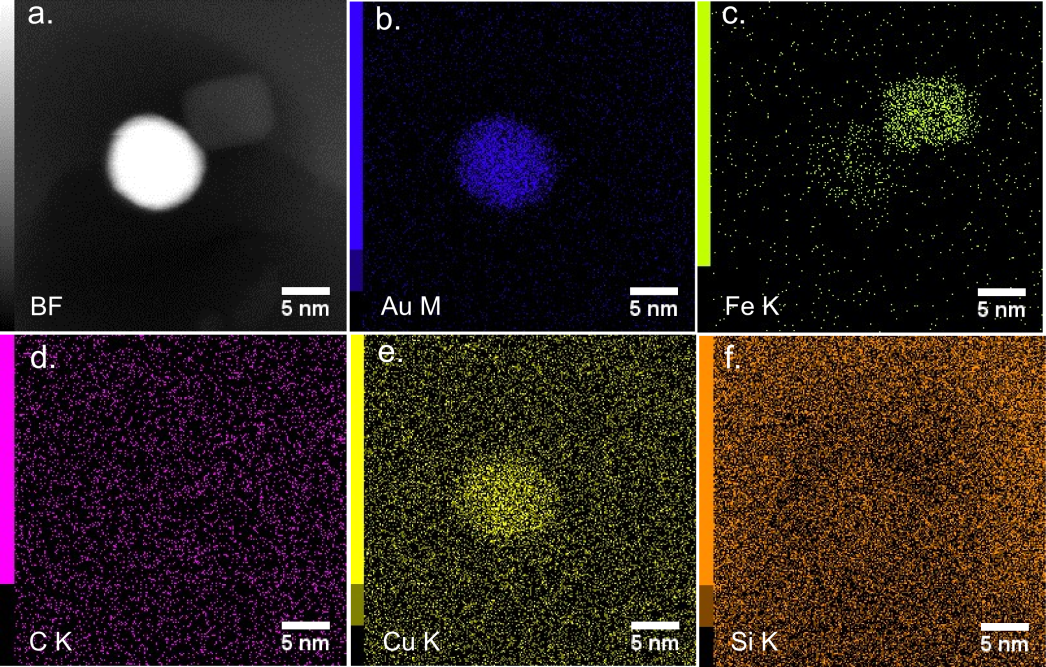 |
| --- |
| **Figure S12:** The elemental mapping of Au- Fe_3_O_4_ heterodimer nanoparticles. (a) The bright field mode image of the corresponding heterodimer; (b) The elemental maps of Au (blue); (c) Fe (green); (d) C (pink); (e) Cu (yellow) and (f) Si (orange). |

1. **Overview TEM micrographs**

**
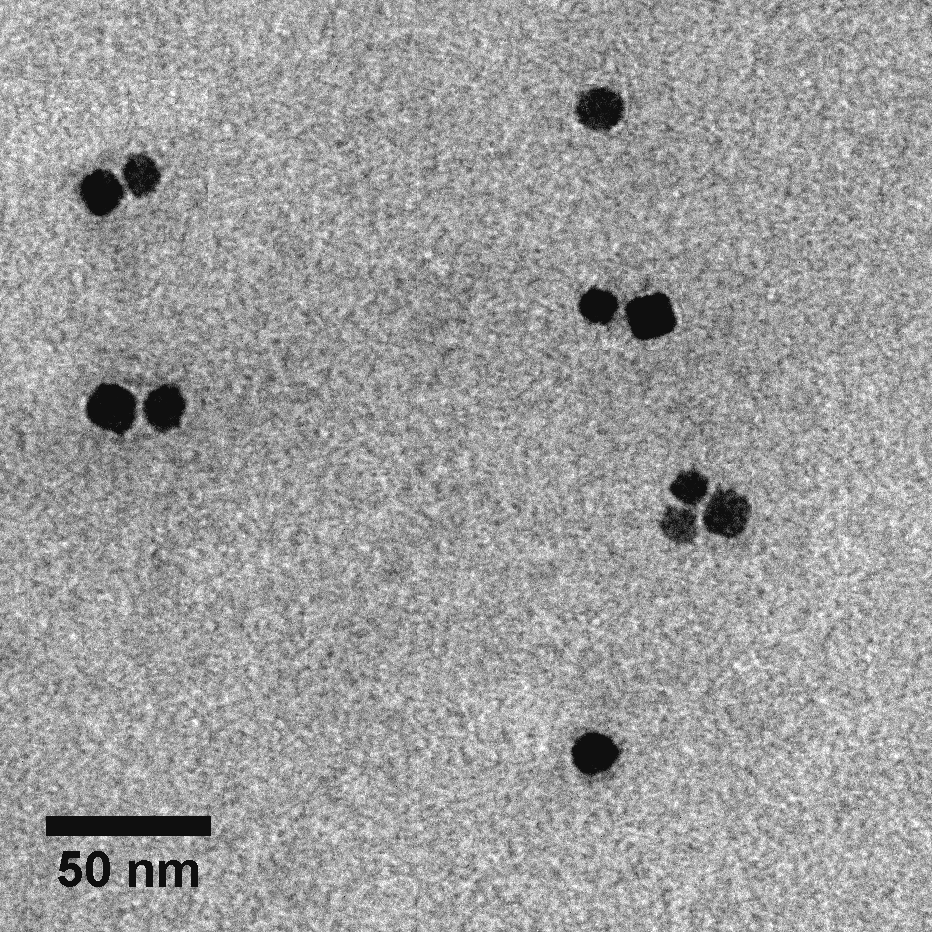
**

|  |  |
| --- | --- |
| **Figure S13**: Overview TEM micrographs of Au/Fe_3_O_4_ hetero-dimers. | |

1. **X- Ray Diffraction**

The crystallographic phase of hetero-dimer nanoparticles was confirmed with X-Ray diffraction (XRD), which showed the simultaneous existence of cubic Fe_3_O_4_ (JCPDS 00-079-1849) and spherical Au (JCPDS 00-004-0784) crystal phases. The XRD patterns are in a good agreement with the selected area electron diffraction (SAED) patterns obtained through TEM.


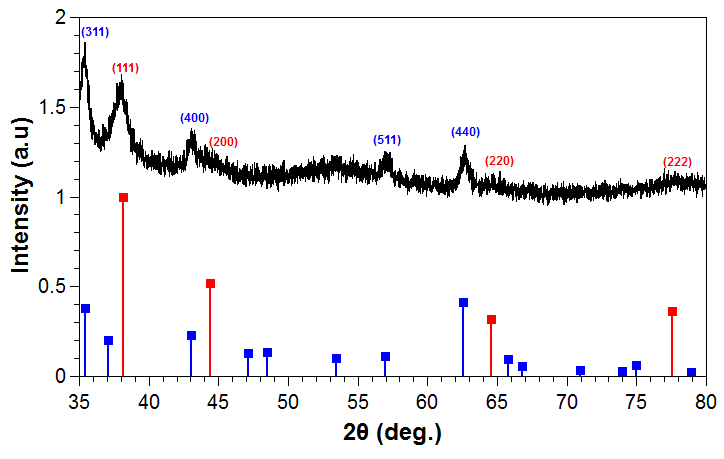


**Fig S14:** XRD pattern showed the crystal phases existence of both Au and Fe_3_O_4_ confirming the successful hetero-dimerisation. *Red* (Au nanoparticles, JCPDS 00-004-0784); *Blue* graph (Fe_3_O_4_, JCPDS 00-079-1849).

1. **UV-Vis**

The optical properties of the Au-Fe_3_O_4_ hetero-dimer nanoparticles were examined by the UV-VIS spectroscopy (cf. Figure 8). The UV-VIS measurements were also utilised to determine the absorption peak of the un-coupled Au and Fe_3_O_4_ nanoparticles for comparison. The UV-VIS characterisation was measured at room temperature and carried out in the 400-800 nm wavelength range. The absorption peak of the Au nanoparticles alone was located at ~521 nm, which represents the surface plasmon resonance (SPR) peak of the Au particles. Pure Fe_3_O_4_ nanoparticles do not exhibit any strong absorption features thus, no significant peaks have been observed. However, the Au-Fe_3_O_4_ hetero-dimer nanoparticles showed an absorption peak at ~517 nm, which could be assigned to the localised SPR. The shift was probably caused by interactions between Fe_3_O_4_ and Au nanoparticles.


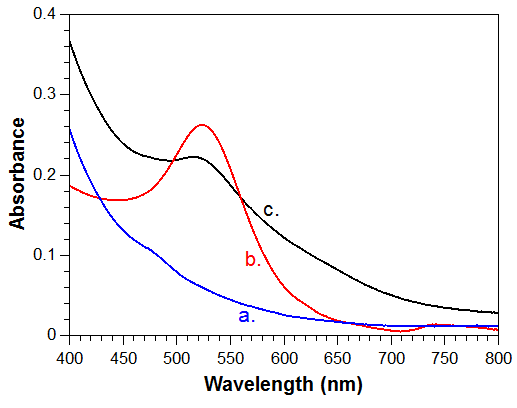


**Fig S15:** The optical absorbance (UV-Vis) spectra of (a) Fe_3_O_4_ NPs; (b) Au nanoparticles; and (c) Au-Fe_3_O_4_ hetero-dimer nanoparticles.

**References**

(1) Dewi, M. R.; Gschneidtner, T. A.; Elmas, S.; Ranford, M.; Moth-Poulsen, K.; Nann, T. Monofunctionalization and Dimerization of Nanoparticles Using Coordination Chemistry. *ACS Nano* **2015**, *9* (2), 1434–1439.

(2) Dewi, M.; Skinner, W.; Nann, T. Synthesis and Phase Transfer of Monodisperse Iron Oxide (Fe3O4) Nanocubes. *Aust. J. Chem.* **2013**.
